# The Promises and Pitfalls of Machine Learning for Detecting Viruses in Aquatic Metagenomes

**DOI:** 10.3389/fmicb.2019.00806

**Published:** 2019-04-16

**Authors:** Alise J. Ponsero, Bonnie L. Hurwitz

**Affiliations:** ^1^Department of Biosystems Engineering, The University of Arizona, Tucson, AZ, United States; ^2^BIO5 Institute, The University of Arizona, Tucson, AZ, United States

**Keywords:** virus, metagenomic, machine learning, sequence classification, viral signature

## Abstract

Tools allowing for the identification of viral sequences in host-associated and environmental metagenomes allows for a better understanding of the genetics and ecology of viruses and their hosts. Recently, new approaches using machine learning methods to distinguish viral from bacterial signal using k-mer sequence signatures were published for identifying viral contigs in metagenomes. The promise of these content-based approaches is the ability to discover new viruses, with no or few known relatives. In this perspective paper, we examine the use of the content-based machine learning tool VirFinder for the identification of viral sequences in aquatic metagenomes and explore the possibility of using ecosystem-focused models targeted to marine metagenomes. We discuss the impact of the training set composition on the tool performance and the current limitation for the retrieval of low abundance viral sequences in metagenomes. We identify potential biases that could arise from machine learning approaches for viral hunting in real-world datasets and suggest possible avenues to overcome them.

## Introduction

Viruses infect host cells from all domains of life and are highly adapted to their host genetics and their environmental niches (Hurwitz et al., 2014; Brum et al., 2015). Recently, metagenomics has laid the groundwork for understanding viruses and their uncultured hosts. Several tools provide rapid and accurate taxonomic assignment of metagenomic sequences directly from a microbiome by comparing them to known bacterial and viral genomes using k-mer based tools, such as Centrifuge (Kim et al., 2016), CLARK (Classifier based on Reduced K-mers) (Ounit et al., 2015), USEARCH (Edgar, 2010), KRAKEN (Wood and Salzberg, 2014), and NBC (Naive Bayes Classifier) (Rosen et al., 2008) [reviewed in Hurwitz et al. (2018)]. Importantly, these tools rely on finding sequence similarity to known viral sequences that represent only a small portion of viral diversity (Roux et al., 2015b). In practice, viromes have a high number of reads with no matches to known viral genomes, in prior studies, less than 10% of reads were assigned from ocean viromes (Hurwitz and Sullivan, 2013).

To explore the viral biodiversity and ecology, a number of bioinformatic tools perform a high-level taxonomic (viral or cellular origin) assignment of metagenomic sequences. They aim to provide means to collect all viral sequences in a metagenome and help the discovery of new viral groups. Several approaches were used: some tools align short reads to a viral marker gene database [MetaPhlAn (Segata et al., 2012), MetaPhlAn2 (Truong et al., 2015)], or to a reference database of whole genomes MG-RAST (Meyer et al., 2008), ViromeScan (Rampelli et al., 2016), VIP (Li et al., 2016), HoloVir (Laffy et al., 2016), FastViromeExplorer (Tithi et al., 2018). Other tools use assembled contigs and align to a viral genome database [Metavir (Roux et al., 2011, 2014), Virome (Wommack et al., 2012), MetaPhinder (Jurtz et al., 2016)].

These reference-based classification tools are limited in their ability to identify novel viruses and are biased toward the identification of previously isolated viruses. However, large scale efforts in retrieving viral sequences in metagenomes and viromes as the IMG/VR database allows for broader research into non-isolated viruses (Paez-Espino et al., 2019). In 2015, the release of VirSorter allows the user to identify potential viral sequences in metagenomes using Hidden Markov Models (HMM). The tool relies on both known viral genomes and viral sequences from viromes for broader detection of unknown viruses (Roux et al., 2015a).

In contrast to these reference-based approaches, an emerging approach is to use composition-based pattern detection leveraging machine learning algorithms. The idea behind this approach is to train a machine learning model to learn to identify a set of features that signal a viral origin to generalize the identification of all viral sequences. VirFinder (Ren et al., 2017) uses a machine learning approach to classify sequences as viral (phages) or prokaryotic based on their k-mer signatures. The model presented in the paper is a logistic classifier, trained on known phages and bacterial genomes from the RefSeq database (we’ll refer to this model as “phages-prok model”), and was shown to provide better accuracy for viral sequence detection than VirSorter, especially on short sequences (<5000 bp). Importantly, the tool was shown to have better recall for the identification of previously unknown phage sequences. The authors also provide a model trained on all DNA viral, including some eukaryotic viruses, and prokaryotic genomes from RefSeq (We’ll refer to this second model as “DNAvirus-prok”). Other machine-learning based tool for viral hunting in metagenome, such as MARVEL were developed (Amgarten et al., 2018; Bzhalava et al., 2018). However, these other tools base their prediction on various genomic features such as the relative synonymous codon usage (Bzhalava et al., 2018), gene density, strand shifts, and the number of significant hits against the pVOGs database (Amgarten et al., 2018). While these approaches are valuable, they lose information contained in non-coding sequences, and their use is limited to long contigs only. Moreover, these tools may add additional bias based on the choice of gene caller for the extraction of the genomic features.

In this work, we review the potential bias and pitfalls of composition-based machine learning approaches such as VirFinder for the detection of viral sequences in aquatic ecosystems. Because MARVEL relies on a pVOGs database, we focused our discussion on VirFinder that to our knowledge is the only tool using a completely database-independent approach for the detection of phages. In particular, we discuss three points of importance: (1) the training set composition of supervised machine-learning models and the possibility to obtain marine-focused models, (2) the impact of eukaryotic contamination in metagenomes and (3) limitations in current tools when considering the low abundance of viral sequences in most metagenomes.

## The Composition of the Training Set and Discovery of New Viruses

Soueidan et al. (2015) explored the difficulties of classification of metagenomic sequences using k-mer-based machine-learning approaches (Soueidan et al., 2015). In their perspective paper, the authors used the concept of “hardness of the task.” Hardness measures were developed to understand why some instances are harder to classify correctly than others (Smith et al., 2014). Overlap of data from different classes was shown to be a principal contributor to instance hardness. Using a *k*-Disagreeing Neighbors (kDN) algorithm, Soueidan et al. (2015) show that, for a k-mer size of 3 bp, the high-level classification of viral sequences mixed with non-viral sequences is a hard task, whereas low-level classification (family level classification) is easier. These results suggest that machine learning models trained to classify viral sequences against cellular sequences may have a hard time generalizing to unknown viral families.

**FIGURE 1:**
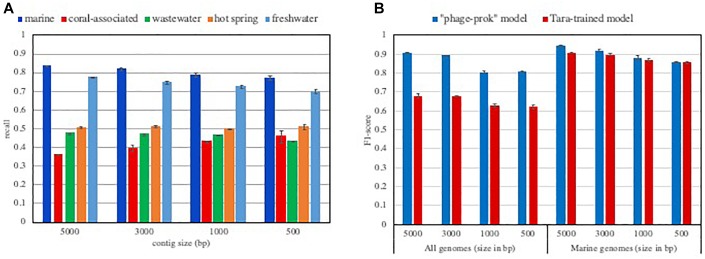
Influence of the training set composition on the model performances. **(A)** Recall of VirFinder “phage-prok” model on viral contigs isolated in various aquatic ecosystems. The recall was assessed for VirFinder “phage-prok” model when considering viral contigs isolated in various aquatic ecosystems (pelagic, freshwater, hot spring, coral-associated and wastewater). The sequences were downloaded from the IMG V/R env database (methods described in Supplementary File S1 and list of metagenomes used in Supplementary File S2). The viral sequences were broken down to 5000, 3000, 1000, and 500 bp and used to evaluate VirFinder “phage-prok” model. The mean of the recall was calculated for three evaluation sets of 2000 viral sequences each with the exception of the coral-associated evaluation sets composed of 200 viral examples due to the low amount of sequence available for this ecosystem. The error bars correspond to the standard deviation on the three measures. **(B)** F1-score of classifiers trained on Tara Oceans Metagenomes. Tara-trained models were trained on 10 000 viral and 10 000 prokaryotic sequences from Tara Oceans metagenomes and viromes broken down to 5000 bp. Previous cleaning steps were performed to ensure a low contamination content of the training set (see Supplementary File S1). The F1-score of a Tara-trained model and of VirFinder’s “phage-prok” model was calculated for evaluation sets composed of viruses and prokaryotes isolated in a marine ecosystem (“marine genomes”) or an evaluation set composed of viral and prokaryotic genomes regardless of their origin (“all genomes”). For the “marine evaluation set,” genomes from phages and prokaryotes isolated in marine ecosystems were downloaded from Genbank and the Patric database, respectively, and the sequences were broken down to 5000, 3000, 1000, and 500 bp (see methods in Supplementary File S1 and list of genomes available in Supplementary File S2). The “all genomes” evaluation set is composed of genomes from phages and prokaryotes from RefSeq database published after 2014 (see methods in Supplementary File S1 and list of genomes available in Supplementary File S2). The mean of the F1-score was calculated for three evaluation sets composed of 2000 viral sequences and 2000 prokaryotic sequences. The error bars correspond to the standard deviation on the three measures.

This idea is further confirmed by the performance of VirFinder that shows a dependence on abundant known viral groups in the tool’s training set. The “phages-prok” model’s viral training set is mainly composed of phages infecting Proteobacteria and firmicutes from the RefSeq database, and on the other hand, the training set is poor in Archaea infecting phages. Discussing this bias in their training set, the authors showed how VirFinder’s performance varied for several groups of viruses. They showed a markedly lower performance for the detection of Archeal phages than Bacterial phages and revealed that the tool is biased toward the identification of the most represented viral groups in their training set (Ren et al., 2017). Because different ecosystems harbor different viral groups and their hosts, we expect VirFinder’s ability to retrieve viral sequences to be significantly affected when considering different ecosystems. We evaluated the true positive rate, or recall (how many truly viral results are returned) from the “phages-prok model” for viral sequences isolated in various aquatic ecosystems (Figure 1A). Each evaluation set was composed of viral sequences isolated in pelagic, freshwater, hot spring, coral-associated and wastewater metagenomes available in the IMG/VR environment database. Not surprisingly, the recall of VirFinder varies according to the considered ecosystem. We measure a lower recall of the tool for viral contigs isolated in hot springs, coral-associated and wastewater environments compared to the tool performance for viral sequences isolated from pelagic and freshwater metagenomes. This suggests that while viral groups present in pelagic ecosystem are now well represented in the RefSeq database, a variety of viruses present in less-studied ecosystems such as coral-associated are currently unavailable (Figure 1A). Some of the differences in recall across the ecosystems can be explained by the presence of sequences from viruses infecting eukaryotes. These sequences would not be recognized as viral by the “phage-prok” model. However, the VirFinder “DNAvirus-prok model,” trained to identify both phage and eukaryote infecting viruses shows the same drop in recall for hot spring, coral-associated and wastewater metagenome (Supplementary Figure S1).

The inability of these models to recognize certain viral groups may be improved by increased sequencing effort of new viral genomes. It indeed is possible to train models on an ever-growing number of sequences: deep learning approaches are particularly suited for this task since they can deal with complex patterns and their performance increases with the number of training examples. In contrast to this approach, we explored into the possibility of training simpler models, tuned to an ecosystem of interest using metagenomic sequences. Indeed, viral communities vary in composition by environment as a function of host populations, which in turn occupy niches defined by specific physical and chemical properties (Hurwitz et al., 2014; Brum et al., 2015). Thus, when working in a given environment, the user only needs to recognize a small subset of viral sequences. Viromes can provide a set of viral signatures from a given ecosystem, that can be used to inform a machine learning model.

As a proof of concept, we developed pelagic-focused classifiers trained on Tara Oceans viromes and microbiomes. Using VirFinder training function, we trained models using metagenomes from the Tara Oceans Dataset (prokaryote-enriched fractions, 0.22 to 1.6 μm, 0.22 to 3 μm) for the non-viral sequences and sequences from the Tara Oceans Viromes (virus-enriched fraction, <0.22 μm) for the viral sequences (Sunagawa et al., 2015) (material and methods are detailed in Supplementary File S1 and a list of metagenomes in Supplementary File S2).

Two evaluation sets were constructed using published phages and prokaryotic genomes, isolated in marine ecosystem (“marine evaluation set”) or isolated in various ecosystems (“all genomes evaluation set”) (material and methods in Supplementary File S1, list of genomes in Supplementary File S2). To ensure that those sequences were not used to train the VirFinder “phages-prok” or “DNAvirus-prok” models, only genomes published after 2014 were used in the evaluation sets.

To take into account both recall and precision (a measure showing how many result returned are truly viral sequences) of the models in this evaluation, the F1-score (harmonic average of precision and recall for the model), was calculated. Globally, the F1-score of Tara-trained models is equivalent to those measured for VirFinder “phage-prok” model on this marine-focused evaluation set. The Tara-trained models also show a preferential detection for marine viral groups as their performance is greatly reduced when sequences from other ecosystems are taken into account (Figure 1B). More precisely, the recall is greatly reduced when the model is evaluated on all RefSeq genomes regardless of their origin, showing an ecosystem-focused specialization of the Tara-trained models (Supplementary Figure S2).

This result shows that it is possible to obtain ecosystem-focused models for the identification of viruses in metagenomes using the information from viromes available for the ecosystem of interest. Although we believe that this approach could be applied to other ecosystems, it is important to highlight that viromes can also provide a biased representation of the actual viral population. For example, viromes uniquely target the dsDNA viral community. Moreover, the DNA extraction method (Wood-Charlson et al., 2015) or the chosen filtration size (López-Pérez et al., 2017) used for viromes can greatly impact the composition of viral group retrieved, and therefore bring a bias in the training set. While this approach could provide an avenue to investigate environments where few viral genomes are available, it requires the availability of several viromes and microbiomes datasets from the ecosystem of interest. Such a sequencing effort is rarely met, however, this issue is expected to be reduced by the increasing number of metagenomic datasets available.

## The Problem of Potential Eukaryotic Contamination

We further argue here that training on an ever-growing number of sequences may lead to unexpected effects. VirFinder published model, “phage-prok” was trained on RefSeq phages and prokaryotic genomes, however, the authors provide online the “DNAvir-prok model,” trained on all DNA viral and prokaryotic genomes from RefSeq. This model is more exhaustive in terms of virus groups included in its training set; however, it shows a strong misclassification of eukaryotic sequences, with an FPR superior 0.7 for genomic sequences from known fungi, plant, human and protozoa (Supplementary Figure S3).

The “phage-prok” model training set does not contain any eukaryotic sequences, and therefore shows an increased false positive rate toward eukaryotic sequences. This false positive rate is further increased when using the “DNAvirus-prok” model, where this misclassification is increased by the sequence length suggesting that this model learned to identify eukaryotic sequences as viral. At a tetra-nucleotide level, prokaryotic and eukaryotic viruses and their hosts have been shown to share a closer sequence composition, providing a potential explanation for this model’s behavior (Pride et al., 2006).

When sequencing a metagenome, eukaryotic contamination is common. Eukaryotic sequences in metagenomes usually come from human contamination when processing the metagenome but can also come from the eukaryotic host when considering host-associated microbiome (human or cow gut metagenome as an example). In those cases, the eukaryotic sequences can easily be removed by mapping the input contigs against the human and host genome. However, in aquatic ecosystems, eukaryotic sequences can also be present in metagenome from micro and pico-eukaryotes naturally present in the ecosystems. Dealing with those sequences is more difficult because of the lack of complete genomes for these organisms in the databases. New tools that take into account eukaryotic sequences are critical for exploring a variety of ecosystems of interest.

## Dealing With Low Viral Content in Metagenomes

While viromes allow for enrichment in viral sequence content, real-world metagenomes often contain a low proportion of viral sequences (Breitbart et al., 2002; Reyes et al., 2010; Daly et al., 2011). Similar to other tools for viral detection in metagenomes, VirFinder’s precision when dealing with rare events is hampered by a simple Bayes’ relationship (Ren et al., 2017). Indeed, when working in datasets where viral reads are rare (less than 10%), the number of false positives can become comparable or even superior to the number of true positive hits. Some metrics for machine learning model performance are appropriate to study such imbalanced datasets. Receiver-operator curves (ROC) are commonly used to evaluate binary classifiers performances. Because ROC curves do not depend on the particular threshold value, they provide a better measure of the tradeoff between true and false positives rates. The area under a ROC curve (AUC) can be used to summarize a model’s performance. It is, however, important to notice that this metric, relies only on true and false positive rates and is therefore misleading when evaluating models on imbalanced datasets. On the other hand, metrics like precision-recall curves (PRC) and the area under precision-recall curves (AUPRC), allows one to measure the loss of precision when moving to imbalanced datasets.

**FIGURE 2:**
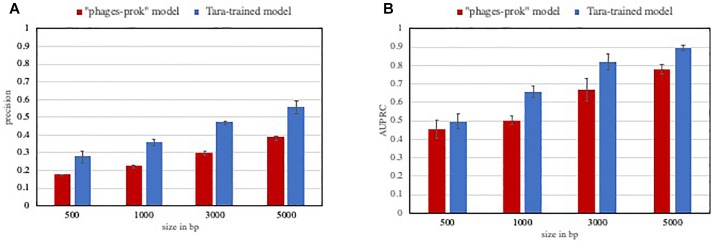
Performance of classifiers on a low viral content evaluation set. The precision **(A)** and Area under the precision-recall curve (AUPRC) **(B)** was calculated for VirFinder “phages-prok” model and a Tara-trained model on an imbalanced marine evaluation set. The evaluation set is composed of sequences from genomes from phages and prokaryotes isolated in marine ecosystems, downloaded from Genbank and the Patric database, respectively, and the sequences were broken down to 5000, 3000, 1000, and 500 bp (see methods in Supplementary File S1 and list of genomes available in Supplementary File S2). The mean precision **(A)** and AUPRC **(B)** was obtained on three evaluation sets composed of 100 viral sequences and 1900 non-viral sequences. The error bars correspond to the standard deviation on these three measures.

In this context, models and methods with increased precision are certainly valuable. As an example, the Tara-trained models showed a lower false positive rate than VirFinder “phage-prok” model on a marine evaluation set. The precision and AUPRC for those models were evaluated on a set composed of 5% viral sequences from marine phage genomes and 95% non-viral sequences from marine prokaryotic genomes. While we do not claim that all ecosystem-focused models would perform better in the detection of rare events, this experiment shows how valuable high precision models can be in the case of very imbalanced datasets, with a significant improvement in the precision (Figure 2A) and AUPRC (Figure 2B) of the Tara-trained model compared to the VirFinder “phage-prok.”

## Discussion

Sample bias occurs when the data used to train the algorithm does not accurately represent the problem space the model will operate in. A model trained on an incomplete and unrepresentative training dataset will be highly unlikely to perform well in real-world situations.

VirFinder is based on a logistic classifier model, trained on genomic datasets from RefSeq. The obtained model is tuned to identify certain viral groups that are well represented in the database. We argue that it is possible to develop ecosystem-focused models that are trained on sequences that are representative of the environment they are specialized in. Because these ecosystem-focused models focus on a subset of viral and prokaryotic groups, they can be trained on a smaller training set than models trying to encompass all ecosystems. As a proof-of-concept, we used metagenomic sequences from the Tara Oceans expedition as training set and obtained models tuned for the identification of marine viral sequences. As expected, our marine-focused models performed poorly on viral groups isolated in other ecosystems. While this approach is limited by the number and quality of viromes available, it is possible that a training set composed of both genomes and sequences from metagenomes could increase the recall of previously uncharacterized viruses.

We strongly believe that the development of machine-learning approaches tuned to deal with low-event detection is the key to developing reliable tools for viral sequence detection in metagenomes. Moreover, the investigation of a large number of ecosystems will require the development of tools dealing with potentially high eukaryotic sequence contamination.

## Author Contributions

BH and AP designed the theoretical study and experiments, analyzed the data, and wrote the manuscript. AP performed the experiments.

## Conflict of Interest Statement

The authors declare that the research was conducted in the absence of any commercial or financial relationships that could be construed as a potential conflict of interest.

## Supplementary Material

FIGURE S1 Recall of VirFinder “DNAvirus-prok” model on viral contigs isolated in various aquatic ecosystems.

FIGURE S2 Recall and precision of a classifier trained on Tara Oceans Metagenomes.

FIGURE S3 False positive rate of VirFinder models for eukaryotic and prokaryotic sequences.

FILE S1 Material and methods.

FILE S2 List of metagenomes and genomes used in training and evaluation sets.
