# Supplementary figures and images for: The Promises and Pitfalls of Machine Learning for Detecting Viruses in Aquatic Metagenomes
Source: Front Microbiol. 2019 Apr 16;10:806. doi: 10.3389/fmicb.2019.00806 (PMC6477088; doi:10.3389/fmicb.2019.00806)

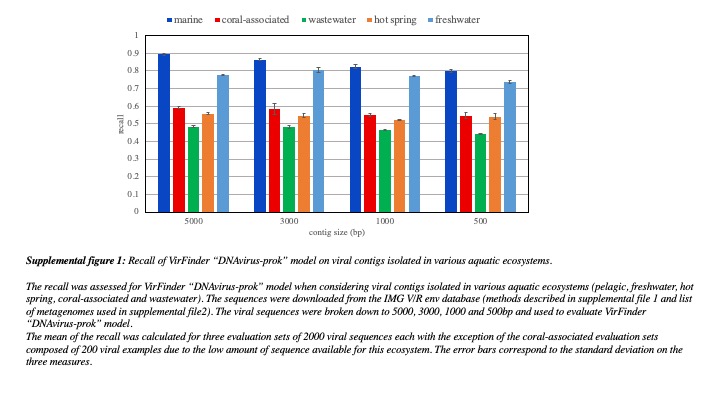

Supplement: FIGURE S1 — Recall of VirFinder “DNAvirus-prok” model on viral contigs isolated in various aquatic ecosystems. [file Image_1.JPEG]

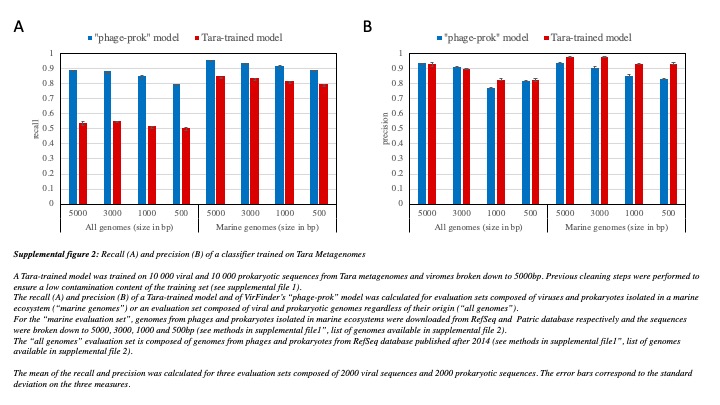

Supplement: FIGURE S2 — Recall and precision of a classifier trained on Tara Oceans Metagenomes. [file Image_2.JPEG]

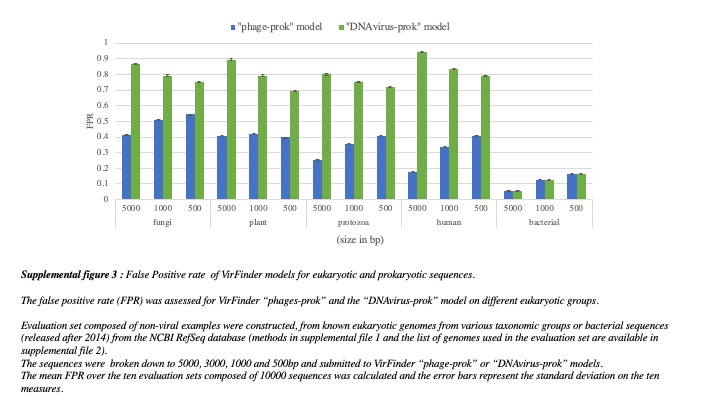

Supplement: FIGURE S3 — False positive rate of VirFinder models for eukaryotic and prokaryotic sequences. [file Image_3.JPEG]
