# Supplementary material for: The Promises and Pitfalls of Machine Learning for Detecting Viruses in Aquatic Metagenomes
Source: Front Microbiol. 2019 Apr 16;10:806. doi: 10.3389/fmicb.2019.00806 (PMC6477088; doi:10.3389/fmicb.2019.00806)
Supplement: FILE S1 — Material and methods. [file Table_1.docx]

Supplemental methods:

**Tara Ocean Metagenomes training set preparation**

Assembled sequences from the Tara Ocean expedition were downloaded from EBI metagenomics (data available at <https://www.ebi.ac.uk/ena/about/tara-oceans-assemblies> list of the metagenomes in Supplemental File2). Contigs with a size less than 5kb were filtered out, and Centrifuge v1.0.4-beta (<https://github.com/infphilo/centrifuge>) was run on the remaining sequences using the Bacteria, Archaea, Human and Virus compressed index (updated 12/06/2016), with the default settings.

Centrifuge is a taxonomic classification engine that enables rapid, accurate, and sensitive labeling of metagenomic sequences. The system uses an optimized indexing scheme that requires a relatively small index and classifies sequences at very high speed (Kim et al., 2016).

Sequences in the microbiomes with viral matches were removed from the negative training set. Sequences in viromes with a Bacterial, Archeal or Human match were removed from the positive training set. The viromes were further cleaned using BLAST against prokaryotic genomes (reference prokaryotic genomes released on ftp://[ftp.ncbi.nlm.nih.gov/blast/db/](http://ftp.ncbi.nlm.nih.gov/blast/db/) last modified on 10/29/18). A cutoff for the e-value was set at 0.01. Sequences with a significant hit against this prokaryotic database were removed from the training set. The remaining sequences were broken down to 5000bp to be used as training set.

**VirFinder training and evaluation parameters**

VirFinder version 1.1 available at <https://github.com/jessieren/VirFinder> was used, and the Tara-trained models were trained using the built-in training function, using a kmer-size of 8bp and a training set of 10 000 viral and 10 000 non-viral sequences randomly selected from the cleaned Tara metagenomic sequences broken down to 5000bp.

A Tara-trained model is available in:

<https://github.com/aponsero/VirFinder_Tara_trained_models>

In this manuscript, we use the “phage-prok model” which is the built-in model for VirFinder, trained on dsDNA phages and prokaryotes genomes from RefSeq published before 2014. “DNAvirus-prok” is a model trained on dsDNA viruses and prokaryotes genomes from RefSeq published before 2014. The model is provided by the authors in GitHub:

<https://github.com/jessieren/VirFinder/raw/master/EPV/VF.modEPV_k8.rda>

**Evaluation sets**

**Set 1- viral sequences from IMG/VR environment database**

Viral sequences identified in aquatic metagenomes were retrieved from the IMG/VR databases (<https://img.jgi.doe.gov/cgi-bin/vr/main.cgi>), using the uncultivated viral genomes study resource (Paez-Espino et al. 2018). Metagenomes from coral-associated, wastewater, hot spring, freshwater, and marine ecosystems were identified using the habitat name (from GOLD) annotation, (list of the metagenomes in supplemental file 2) and identified viral contigs from those studies were downloaded.

Sequences shorter than 5000bp were filtered out and the remaining contigs were broken down to 5000bp, 3000bp, 1000bp, and 500bp. 2000 or 200 sequences were randomly taken from this set to create the three evaluation sets.

**Set 2- “Marine” evaluation set**

Phages complete and partial genomes from GenBank database isolated in a marine ecosystem were identified and downloaded. These genomes were obtained from various marine samples during the CAMERA project “Moore marine phages” (list of genomes in supplemental file2).

Bacterial and archaeal complete and partial genomes isolated in marine ecosystem were downloaded from the PATRIC database (list of genomes in supplemental File2).

Sequences were broken down to 5000bp and the prokaryotic genomes were searched for potential prophage sequences using BLAST against viral genomes (RefSeq DNA viral complete genomes, last updated January 2018). A cutoff for the e-value was set at 0.01. Any prokaryotic sequence with a significant match to this viral database was removed from the evaluation set.

The remaining sequences were broken down to 5000bp, 3000bp, 1000bp and 500bp. To create the evaluation sets 2000 viral and 2000 prokaryotic sequences (50% / 50% evaluation set) or 100 viral and 1900 prokaryotic sequences (5% / 95 % evaluation set), were randomly sampled from the sets.

**Set 3- “All genomes” evaluation set**

Phage complete genomes from RefSeq database published after 2014 were identified and downloaded (list of genomes in supplemental file2).

Bacterial and archaeal complete genomes released after 2014 were downloaded from the RefSeq database (list of genomes in supplemental File2).

Sequences where broken down to 5000bp and the prokaryotic set was searched for potential prophage sequences using BLAST against viral genomes (RefSeq DNA viral complete genomes, last updated January 2018). A cutoff for the e-value was set at 0.01. Any sequences from the prokaryotic set with a significant hit to the viral database were removed from the evaluation.

The remaining sequences were broken down to 5000bp, 3000bp, 1000bp and 500bp. Evaluation sets were obtained by randomly sampling 2000 viral and 2000 prokaryotic sequences.

**Set 4- Eukaryotic sequences**

Complete and partial genomes available from the RefSeq database were downloaded for fungi, plants, and protozoan (list of genomes in supplemental File2). The human genome GRCh38 (Genome Reference Consortium Human Build 38) was downloaded from RefSeq (Assembly accession GCF_000001405.26).

Sequences shorter than 5000bp were filtered out and the remaining contigs were broken down to 5000bp, 1000bp, and 500bp. The evaluation sets were obtained by randomly sampling 10000 sequences for each group.

**Evaluation measures**

For all the studied models, when evaluating the Recall, Precision or F1-score, we define a “positive hit” (sequence predicted as viral) as a hit with a score strictly above 0.5. On the contrary, a negative hit (sequence predicted as non-viral) is defined as a hit with a score equal or below 0.5.

The models performance were assessed using custom scripts to calculate the following metrics :

(available in <https://github.com/aponsero/VirFinder_Tara_trained_models/tree/master/scripts> )

| **Metric name** | **formula** |
| --- | --- |
| Recall (R) also called True positive rate | R=Tp/(Tp+Fn) |
| Precision (P) | P=Tp/(Tp+Fp) |
| F1-Score (F1) | F1=2((P*R)/(P+R)) |
| False positive rate (FPR) | FPR= Fp/(Fp+Tn) |

Tp= True positive; Fn=False negative; Tn=True negative; Fp=False positive

AUPRC (Area under the precision-recall curve) was assessed on the models using the python library SciKit learn:

<https://scikit-learn.org/stable/auto_examples/model_selection/plot_precision_recall.html#sphx-glr-auto-examples-model-selection-plot-precision-recall-py>

Precision-Recall is a useful measure of the success of prediction when the classes are very imbalanced, and the precision-recall curve shows the tradeoff between precision and recall for the different thresholds.

A high AUPRC proves that the model has high recall and high precision. A high score, therefore, shows that the classifier is returning accurate results (high precision), as well as returning mostly relevant results (high recall).

**References**

Kim, D., Song, L., Breitwieser, F. P., and Salzberg, S. L. (2016). Centrifuge: rapid and sensitive classification of metagenomic sequences. *Genome Res.* doi:10.1101/gr.210641.116.

Paez-Espino, D., Roux, S., Chen, I.-M. A., Palaniappan, K., Ratner, A., Chu, K., et al. (2018) IMG/VR v.2.0: an integrated data management and analysis system for cultivated and environmental viral genomes. *Nucleic Acids Res.* doi:10.1093/nar/gky1127.
